# Optimization the Process of Chemically Modified Carbon Nanofiber Coated Monolith via Response Surface Methodology for CO_2_ Capture

**DOI:** 10.3390/ma13071775

**Published:** 2020-04-10

**Authors:** Mohamad Rasool Malekbala, Soroush Soltani, Suraya Abdul Rashid, Luqman Chuah Abdullah, Umer Rashid, Imededdine Arbi Nehdi, Thomas Shean Yaw Choong, Siow Hwa Teo

**Affiliations:** 1 Department of Chemical and Environmental Engineering, Universiti Putra Malaysia, Selangor 43400, Malaysia; m.r.malekbala@gmail.com (M.R.M.); soroush.soltaani@gmail.com (S.S.); chuah@upm.edu.my (L.C.A.); 2 Materials Processing and Technology Laboratory, Institute of Advanced Technology, Universiti Putra Malaysia, Serdang 43400, Selangor, Malaysia; suraya_ar@upm.edu.my; 3 Institute of Advanced Technology, Universiti Putra Malaysia, Selangor 43400, Malaysia; umer.rashid@upm.edu.my; 4 Department of Chemistry, College of Science, King Saud University, Riyadh 11451, Saudi Arabia; imed12002@gmail.com; 5 Laboratoire de Recherche LR18ES08, Chemistry Department, Science College, Tunis El Manar University, Tunis 2092, Tunisia; 6 Chancellery Office, Universiti Malaysia Sabah, Kota Kinabalu 88400, Sabah, Malaysia; siowhwa_teo@hotmail.com

**Keywords:** carbon nanofiber (CNF), monolith substrate, catalyst promoter, response surface methodology (RSM), CO_2_ adsorption

## Abstract

In the present study, a sequence of experiments was performed to assess the influence of the key process parameters on the formation of a carbon nanofiber-coated monolith (CNFCM), using a four-level factorial design in response surface methodology (RSM). The effect of reaction temperature, hydrocarbon flow rate, catalyst and catalyst promoter were examined using RSM to enhance the formation yield of CNFs on a monolith substrate. To calculate carbon yield, a quadratic polynomial model was modified through multiple regression analysis and the best possible reaction conditions were found as follows: a reaction temperature of 800 °C, furfuryl alcohol flow of 0.08525 mL/min, ferrocene catalyst concentration of 2.21 g. According to the characterization study, the synthesized CNFs showed a high graphitization which were uniformly distributed on a monolith substrate. Besides this, the feasibility of carbon dioxide (CO_2_) adsorption from the gaseous mixture (N_2_/CO_2_) under a range of experimental conditions was investigated at monolithic column. To get the most out of the CO_2_ capture, an as-prepared sample was post-modified using ammonia. Furthermore, a deactivation model (DM) was introduced for the purpose of studying the breakthrough curves. The CO_2_ adsorption onto CNFCM was experimentally examined under following operating conditions: a temperature of 30–50 °C, pressure of 1–2 bar, flow rate of 50–90 mL/min, and CO_2_ feed amount of 10–40 vol.%. A lower adsorption capacity and shorter breakthrough time were detected by escalating the temperature. On the other hand, the capacity for CO_2_ adsorption increased by raising the CO_2_ feed amount, feed flow rate, and operating pressure. The comparative evaluation of CO_2_ uptake over unmodified and modified CNFCM adsorbents confirmed that the introduced modification procedure caused a substantial improvement in CO_2_ adsorption.

## 1. Introduction

As an atmospheric greenhouse gas (GHG), carbon dioxide (CO_2_) is considered as one of the most vital contributors to global warming. It is highly essential to reduce the total emissions of CO_2_, for example through the substitution of fossil fuels with renewable energy sources, to limit the average global temperature growth to 2 °C [1]. At present, adsorption is known as a practical procedure to capture CO_2_ on an industrial scale, in which adsorbates typically attach to the surface of an adsorbent [2,3]. A crucial requirement in the economic separation procedure is to apply adsorbents with a satisfactorily high selectivity, capability, and reaction time [4]. To date, a broad range of adsorbents, such as zeolites, metal organic structure, carbonic materials, and polymeric composites, have been used for the adsorption of CO_2_ [5,6,7].

The adsorption system and adsorbent’s effectiveness can be simply determined by the surface chemistry of the porous structure. The adsorption quality can be typically accessed by the adsorbent surface properties (spacing, pore size, and polarity) and the adsorbed components’ characteristics (polarity, molecular mass and dimension). Knowing these special physical and textural features of the adsorbed particles and the adsorbent surface can ease the separation of CO_2_ from a mixture of gases. As an exothermic procedure, the renewal of the adsorbents over desorption could be done via increasing the operating temperature. Nevertheless, the adsorption procedure requires less energy compared with the absorption procedure [8].

Recently, a wide range of sophisticated carbon-based nanomaterials like graphene, carbon aerogel, fullerene, carbon nanotubes (CNTs), and carbon nanofibers (CNFs) have drawn attention due to the adsorbent’s efficiency [9,10]. The CNFs with unique structural, textural, and thermal characteristics can be fabricated using various techniques consisting of laser ablation, arc discharge, and catalytic chemical vapor deposition (CCVD), [11,12,13,14]. Amongst the known fabrication approaches, CCVD is highly preferred due to its remarkable advantages such as high purity, growth control, easy industrialization, and low production cost [15]. To gain a successful growth of CNFs using the CCVD technique, several key factors, including reaction temperature, catalyst concentration, hydrocarbon and carrier gas flow rates, substrate and synthesis time, should be considered [16]. Therefore, strict control of these variables is highly recommended during synthesis of the carbon nanofiber-coated monolith (CNFCM) over the CVD technique.

It has been previously reported that a ferrocene catalyst can effectively enhance the expansion of CNFs on the monolith substrates by furfuryl alcohol decomposition. The first aim of the current research was optimizing the process of CNFs’ coating growth on monolith substrates by tuning the reaction terms. As well as this, the importance of various reaction factors on the synthesis and coating of CNFs on monolith substrates were studied.

Response surface methodology (RSM) is a pragmatic statistical approach to detect the greatest deterioration model and reaction conditions [17] of the experiment. This method uses a factorial design to obtain a make statistical models that reveal the importance of different parameters on the response. This method is a great approach for experiments including multi-parameters and possesses the benefit of identifying superior reaction conditions via showing a general association among different parameters. In this research, the four-level factorial plan in response surface methodology (RSM) was employed in order to identify the key important factors on fabrication procedure. The results were systematically analyzed via variance (ANOVA) in Design Expert (version 10.0) analysis. Furthermore, the capacity and adsorption rates of CO_2_ on CNFCM was examined over a variety of reaction conditions including operating temperature of 30–50 °C, flow rate of 50–90 mL/min, pressure of 1–2 bar, and CO_2_ concentration of 10–40 vol.%. Later, the breakthrough curves for each reaction condition were analyzed.

## 2. Experimental

### 2.1. Materials

Cordierite monoliths (2MgO·2Al_2_O·5SiO_2_) were supplied by Beihai Haihuang Chemical Packing Co.Ltd., Beihai, China with cell compactness of 400 cells per square inch and the dimension of 20 (D) × 25 (L) mm^2^. Furfuryl alcohol [C_5_H_6_O_2_], Aluminium sulfate hexadecahydrate [Al_2_(SO_4_)_3_·16H_2_O], and ferrocene [Fe(C_5_H_5_)_2_], were provided by Sigma-Aldrich, Petaling Jaya, Malaysia. Purified gases of CO_2_ (99.99%), N_2_ (99.99%), Ar (99.99%), and H_2_ (99.99%) were purchased from Linde AG Company, Serdang, Selangor, Malaysia. Ammonia (NH_3_, 65%), was obtained from Chemical Packing Co. Ltd., Beihai, China. 

### 2.2. Synthesis of CNFCM

In this work, the CNFs was formed over a monolith substrate by decomposition in the presence of a catalyst promoter. Initially, 400 cpsi cordierite monolith was wash-coated using aluminum sulfate and then calcination for 3 h at 900 °C. Next, the wash-coated monolith was coated with iron nitrate as a catalyst promoter and then transferred into a tube furnace reactor calcinated at 500 °C for 2 h under air atmosphere to get rid of any outstanding volatiles. The furnace temperature was raised to the growth temperature (700–900 °C) for 2 h. The mixture of furfuryl alcohol/ferrocene was fed with the mixture of 50/50 vol.% H_2_/Ar into the vaporizer zone at 700 °C. The vaporized mixer with H_2_ and Ar was continuously introduced into the tubular quartz. It should be noted that the ferrocene/furfuryle alcohol ratio was 18.41 wt./vol.%. After 120 min, the oven was cooled down to room temperature over an Ar flow rate of 10 mL/min. Finally, the CNFCM composite was taken off from the tube furnace reactor for further examination.

### 2.3. Post-Modification of the Adsorbent

The post-modification was performed using ammonia where 50 mL of the concentrated NH_3_ was mixed with 1000 mL distilled water (DW). Then, 0.7 g of as-prepared CNFCM was added to a capped glass bottle containing 200 mL NH_3_ solution. The mixture was later positioned in a thermo-stated bath for 36 h, and then the samples were removed from the shaker and washed a few times with DW. Next, the washed CNFCMs were dried at 100 °C in an electrical oven for 24 h. The dried samples were labeled as modified-CNFCMs (MCNFCM).

### 2.4. Characterization Methods

The Brunauer Emmet and Teller (BET) model was applied in order to assess the textural properties of the synthesized CNFCM composites, using Micromeritics Tristar apparatus (Brussels, Belgium). The principle of BET is basically founded on the adsorption–desorption of nitrogen, at −196.15 °C, into the surface of CNFCM composites under van der Waals forces. The Barret–Joyner–Halenda (BJH) method was applied to ascertain the porosity of the synthesized CNFCM composites. Prior to BET, the pre-treatment process was started through the elimination of water and degassing of CNFCM composites by pre-heating at 200 °C for 120 min. 

Raman spectroscopy was applied to investigate the structural features of the synthesized CNFCM composites, using a PerkinElmer GXFT.IR, Shizuoka, Japan; Raman spectrometer fitted with an NdYAG laser (λ_0_ = 1064 nm).

The microscopic morphology of the synthesized CNFCM composites was evaluated via field emission scanning electron microscopy (FE-SEM; Sirion-100, Hillsboro, OR, USA) and transmission electron microscopy (TEM; Philips CM-12, MA, USA). 

The carbon yield of the CNFCM composites was later calculated via the following Equation [10]

(1) $$\mathrm{CNF}\;\left(\mathrm{wt}.\%\right)=\frac{m_{f}-m_{i}}{m_{i}\times 100}$$

where m_f_ is the overall accumulation gained after the ending of the course of action, and m_i_ is the accumulation of the wash-coated monolith [10].

### 2.5. CO_2_ Adsorption Process

**Figure 1:**
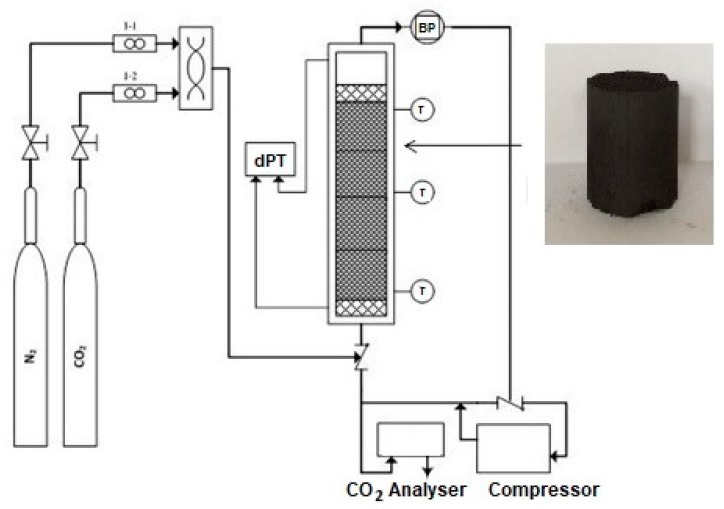
A graphical illustration of the experimental set-up for adsorption of carbon dioxide (CO_2_).

A jacketed stainless-steel cylinder with a 20-cm-long, internal diameter of 1.5 cm and wall thickness of 0.3 was utilized as the adsorption column which was filled with about 2 g of CNFCM. The gas multiple structures included two outlines fixed with two mass-flow controllers (Bronkhorst High-Tech, Shanghai, China), which were utilized to set up a blend of N_2_/CO_2_ to the column. The N_2_ and CO_2_ gas flow rates were attuned prior to inflowing the column to achieve an invariable entire gas flow within 50–90 mL/min over the entire procedure. A gas analyzer (GFG; CO_2_; 50%) was utilized to observe the CO_2_ absorption at the bed outlet. The temperature sensors, with a precision of ± 1.50 °C, were coupled to the body of the column for measuring of the temperature. The adsorption column was then covered with insulator to preserve stable temperatures where an electrical heating jacket was inserted to the set-up to tune and to preserve the preferred temperature in the column. Then, the column was pressurized with inert N_2_ gas to conduct the CO_2_ breakthrough studies. The composition of CO_2_ was constantly monitored in the effluent gas column, as a function of time, until the same concentrations of outlet and inlet CO_2_ were observed. It showed dissemination terms (*C*/*C_o_* = 0.99) where zero CO_2_ can be adsorbed in the column. The adsorbed CO_2_ was fully desorbed through exclusion with the column with N_2_, whilst the temperature of the column kept at 80 °C. It should be noted that the process of adsorption was reversible upon 80 °C, thus allowing for the adsorbent reprocess. A graphical illustration of the experimental set-up is displayed in Figure 1.

The adsorption capability and energetic performance of the bed were assessed as a role of the feed absorption, gas flow rate and adsorbent’s mass. The adsorption capability of the fabricated N_2_-enriched gel beads for CO_2_ was determined via Equation (2) [18,19]

(2) $$q=C_{0}F/m\int_{0}^{t_{b}}\left(1-C/C_{0}\right)dt$$

where *F*, *m*, *C_0_* and *t_b_* are volumetric flow rate of gaseous blend (mmol/min), amount of the adsorbent (*q*), CO_2_ absorption (vol.%) and the breakthrough time (*t*), respectively. *C_0_* and *C* are the inlet and effluent CO_2_ absorption (vol.%), respectively. The breakthrough time was corrected through operating blank experiments in the fixed bed at the same flow rate, pressure, and temperature, conditions in the absence of an adsorbent.

### 2.6. Renewal Experiments

By the end of the adsorption cycle, the temperature of the column was raised to 80 °C where the renewal procedure was aided via a compressor at the last fraction of the column. For CNFCM and the modified-CNFCM, the adsorption procedure was carried out at a lower reaction temperature and a higher pressure.

For the desorption procedure, the operating temperature of 80 °C was selected for each reaction run. By the eradication of N_2_ into the fixed bed, the adsorbed CO_2_ was desorbed from the adsorbent. At the same time, the absorption of the effluent gas was constantly checked via online monitoring using a CO_2_ analyser. The progression was stopped as soon as the CO_2_ analyser indicator showed zero value. Repetitive adsorption/renewal reactions revealed the extraordinary constancy of the separation progression of the adsorbent.

### 2.7. Model Description

A deactivation model (DM) was proposed for the forecast of breakthrough curves in packed columns [18,19]. The prediction model was highly in accordance with experimental data for CO_2_ and H_2_S breakthrough curves. The DM was subjected in view of adsorption activities to adjust the breakthrough curve of the adsorption progression. The DM for CO_2_ adsorption was formulated using assumptions of the iso-thermal structure, pseudo-steady state, mass-transfer strength, insignificant axial distribution, and first-order reaction (adsorption), considering the deactivation of the solid surface for the adsorbent. Assuming the concentration to be self-determining, Equations (3) and (4) were introduced [18,19]

(3) $$C/C_{0}=exp⎣-k_{0}\left(S_{0}/Q_{0}\right)\exp(-k_{d}t)⎦$$

(4) $$\propto =\left[S_{0}/Q_{0}\right]$$

Replacing both Equations (3) and (4), will give Equation (5) upon rearrangement:

(5) $$\ln\left(\ln\left(C/C_{0}\right)\right)=\ln\left(k_{0}\alpha \right)-k_{d}t$$

where the *k_0_*, *k_d_* and *t* parameters are the surface reaction rate constant, first-order deactivation rate index, and operating time, respectively. The surface times (*α*) equal the ratio of the adsorbent surface area over the volumetric flow rate of the gas. The average amount of three experimental records was calculated to lessen the virtual errors (5%). The adsorption quantity of the adsorbents was determined using the adsorption breakthrough curves. For each reaction run, the adsorbed sum of CO_2_ on the samples was gained through the breakthrough curve as in Equation (6)

(6) $$q_{t}=FC_{0}/W\times t_{b}$$

where *W* is the sum of the adsorbent (*q*).

## 3. Results

### 3.1. RSM Modeling and Optimization

The RSM experimental design was performed at 3 levels (+1, 0, −1) with a four-level factorial design including reaction temperature, hydrocarbon flow rate, catalyst and catalyst promoter, indicated in Table 1. A total of 29 reactions were done to assess the impact of different key elements on the expansion of the CNFCM. The effect of vital process elements on the yield of the CNFCM was calculated using the software design expert. This software aided in performing all necessary estimations and determining the importance level of all parameters. The responses from the resulting 29 runs were analyzed via introducing the coefficient of determination (R^2^), analysis of variance (ANOVA), and response plots, as indicated in Table 2.

Table 3 indicates the response data, including linear, two-factor-interaction (2FI), quadratic and cubic polynomial models, using experiment software. According to the results, the linear, 2FI and quadratic models were noteworthy due to their possessing probability values “Prob > F” of below 0.05. An assessment of R^2^ showed that the linear model possessed an undesirable R^2^ value of 0.6026, and therefore it was not considered for further examinations. The quadratic model was preferred according to the following norms: ***(i)*** the “Prob > F” value poorer than 2FI projected model and ***(ii)*** R^2^ value greater than 0.9181.

### 3.2. Statistical Analysis and Modelling

The analyses of variance (ANOVA) for the carbon yield responses are outlined in Table 4. The “Prob > F” values proved the implication of each term in the ANOVA. The values of “Prob > F” less than 0.05 proved the noteworthy model conditions. In this case, the effect of operating temperature (A), ferrocene catalyst amount (B), furfuryl alcohol flow-rate (C), the second-order effect of reaction temperature (A2), and the interface consequence of furfuryl alcohol flow rate with reaction temperature (AC) were considered in turn and emerged as the key determinants of carbon yield. The values larger than 0.05 showed that model conditions had a negligible impact on the carbon yield. It should be noted that these terms were not removed from the analysis to guarantee hierarchical steadiness of the selected quadratic model.

**Figure 2:**
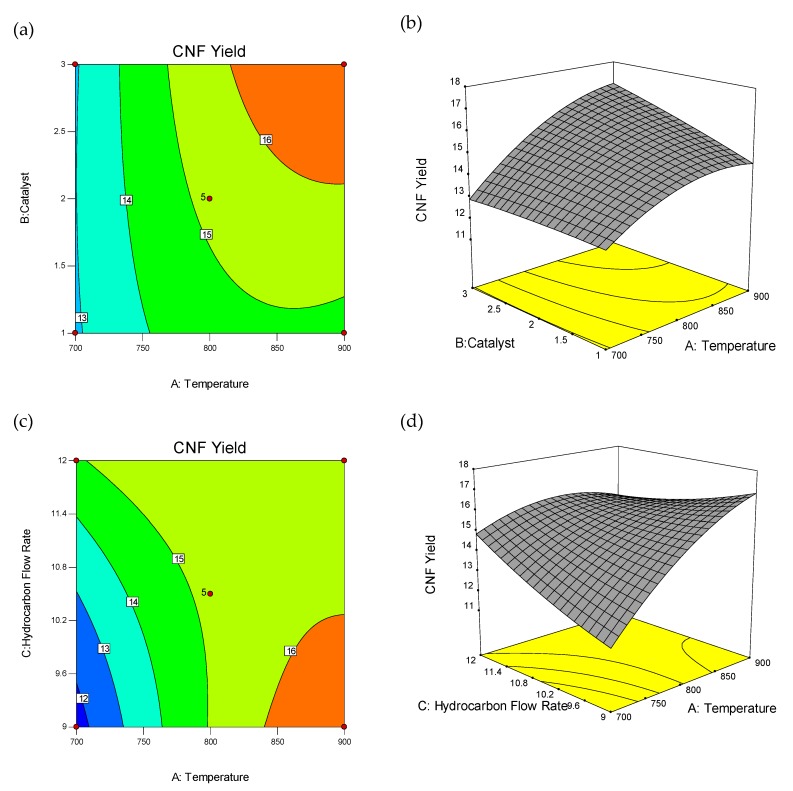

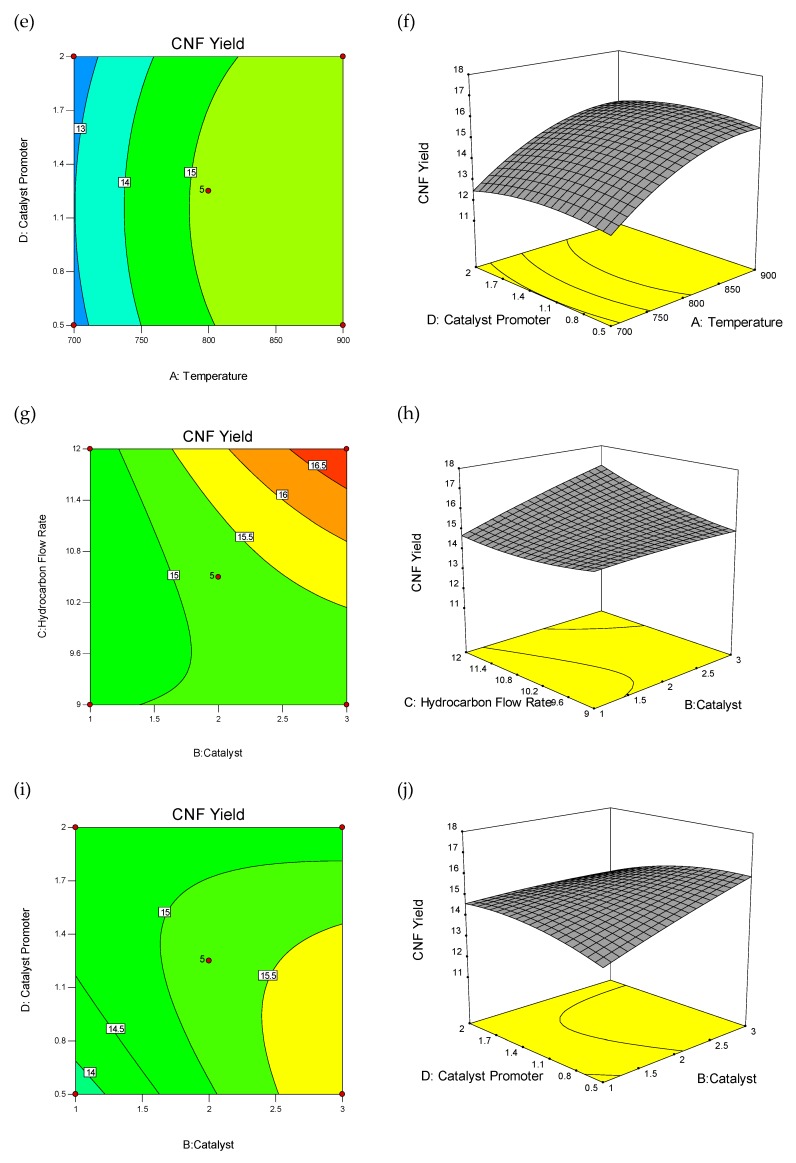

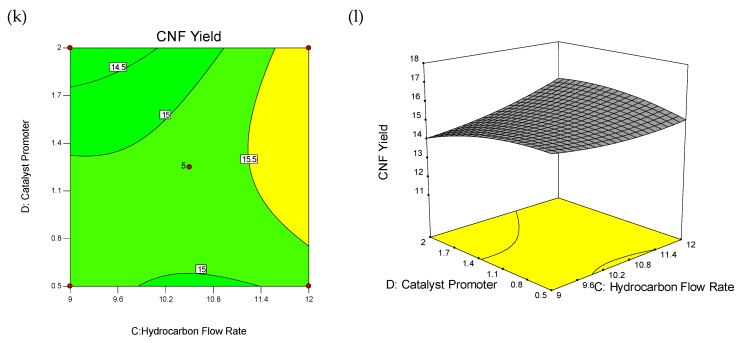
3D wireframe surface plot and contour plot: Interaction effect of factors on carbon nanofiber (CNF) yield (%). Contour Plot of Interactions between Temperature and Catalyst Weight (**a**), Temperature and Hydrocarbon Flow Rate (**c**), Temperature and Catalyst Promoter Concentration (**e**), Hydrocarbon Flow Rate and Catalyst Weight (**g**), Catalyst Promoter Concentration and catalyst weight (**i**), Catalyst Promoter Concentration and Hydrocarbon Flow Rate (**k**), and 3D Wireframe Surface Plot of the Interactions between Temperature and Catalyst Weight (**b**), Temperature and Hydrocarbon Flow Rate (**d**), Temperature and Catalyst Promoter Concentration (**f**), Hydrocarbon Flow Rate and Catalyst Weight (**h**), Catalyst Promoter Concentration and Catalyst Weight (**j**), Catalyst Promoter Concentration and Hydrocarbon Flow Rate (**l**) on Yield of CNF.

The wire-frame 3D plot and interaction effect of factors on CNFs yield (%) is shown in Figure 2, as the probable curvature and its amount could be observed. The response surface could be examined via projecting its surface onto a plane surface beneath. In contour plots, lines of invariable response were drawn on an x–y plane with an axis corresponding to a one factor-level. It should be noted that each line cleanly shows a particular altitude in the surface diagram. The key lead of contour plots was its potential to explore the grade of factors which may result from changing the height/outline of a response surface. 

The regression equation, which was constructed based on the actual factors for carbon yield (%), was calculated via Equation (7):

Carbon yield (%) = 15.22 + 1.45A + 0.59B + 0.43C − 0.11D + 0.56AB − 1.2AC − 9.11AD + 0.53BC − 0.55BD + 0.47CD − 0.79A2 − 0.08B2 + 0.27C2 − 0.39D2(7)

**Figure 3:**
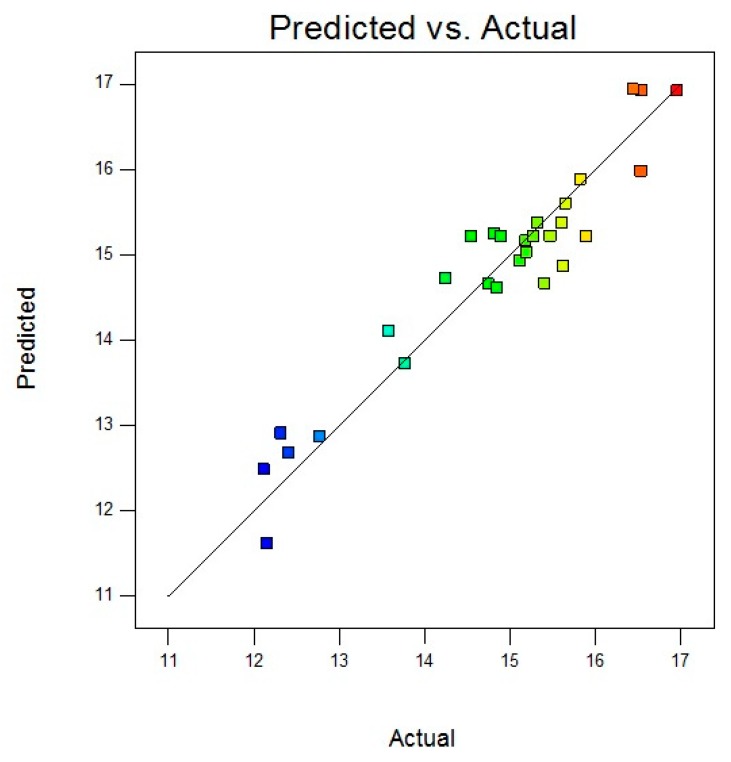
Correspondence plot of predicted and actual values of CNF yield (%).

As demonstrated in Figure 3, the virtual model was highly in accordance with the obtained experimental results, indicated by its high R^2^ value (R^2^ = 0.911).

### 3.3. Optimization Study

The individual parameters and the responses believed to be vital in the fabrication of the CNFCM were simultaneously optimized using a mathematical optimization approach in the Design Expert software (Table 5). It is worth considering that all the parameters and responses relating to the upper/lower restrictions of the reaction scope have to fulfill the norms specified for the best possible operating conditions. The principal was to effectively maximize the growth rate of the CNFCM. There were several predicted sets to achieve the optimum terms, organized by appropriateness. The optimal reaction restrictions for the ultimate suitability were found to be as follows: a reaction temperature of 800 °C, ferrocene catalyst amount of 2.21 g, catalyst promoter amount of 0.095 g/mL, and furfuryl alcohol flow-rate of 0.08525 mL/min.

**Figure 4:**
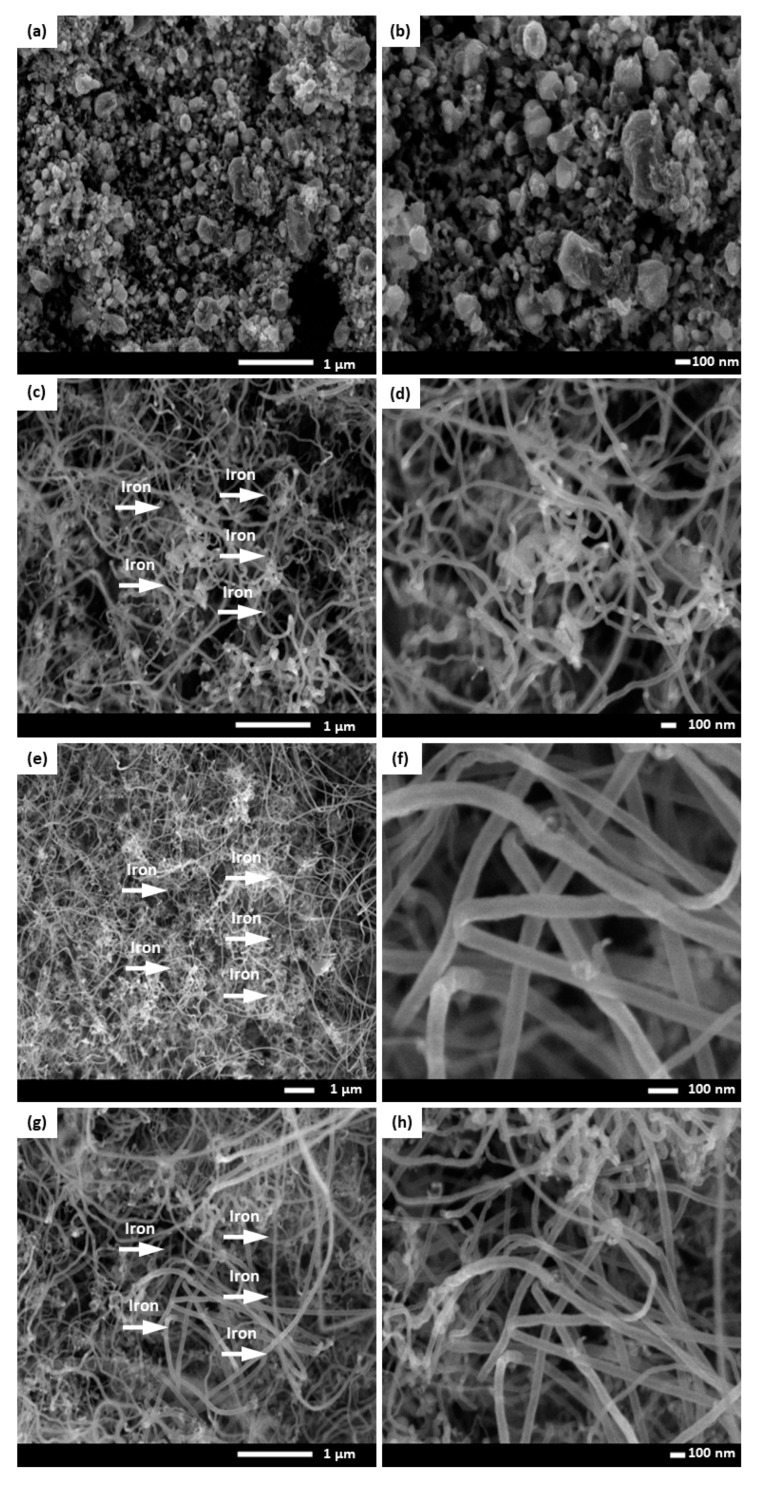
FE-SEM images of the carbon nanofiber-coated monoliths (CNFCMs) synthesized at various temperatures (**a** and **b**) 600 °C, (**c** and **d**) 700 °C, (**e** and **f**) 800 °C, (**g** and **h**) 900 °C. Red arrow: iron catalyst.

**Figure 5:**
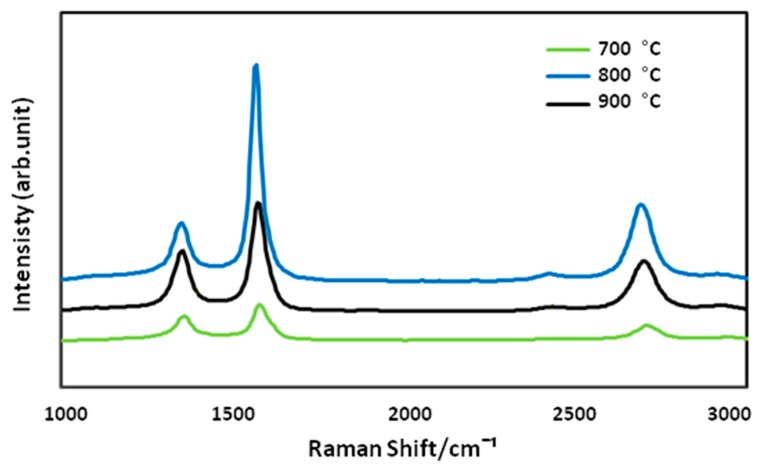
Raman spectra of the CNFCMs synthesized at various temperatures.

The effect of four different reaction temperatures (600, 700, 800 and 900 °C) on the growing rate of optimized CNFCM were further studied. It was observed that the reaction temperature had a great effect on the CNF growth, which was highly in accordance with the RSM results. As can be seen in Figure 4, optimized CNFs started coating on the monolith surface only at the temperature of 700 °C. Due to the extremely low activity of the catalyst at below 700 °C, shapeless carbon formed on the monolith surface, resulting in defective structures of the CNFCM (Figure 4a,b). This revealed that the graphitization of the carbon nanoparticles can only take place at reaction temperatures well above 700 °C. The raman spectra at 700 °C indicated that the D and G peaks possessed very low intensity as compared to the other temperatures. This might correspond to the presence of unstructured carbon on the monolith surface. By raising the temperature from 700 to 900 °C, the graphitization of the CNFs took place, resulting in the higher growth rate of the CNFCM (Figure 4c–f). According to the Raman spectra of the optimized CNFs coated on the monolith surface (Figure 5), carbon nanoparticles formed at 800 °C possessed a higher degree of graphitization, which confirmed the existence of CNFs. Therefore, the most favorable operating temperature was found to be at 800 °C to develop CNFs of high quality and purity. However, further increasing the reaction temperature is not recommended, as extending the reaction temperature to 900 °C may cause deactivation of the catalyst [20].

### 3.4. Effect of Ferrocene Concentration

**Figure 6:**
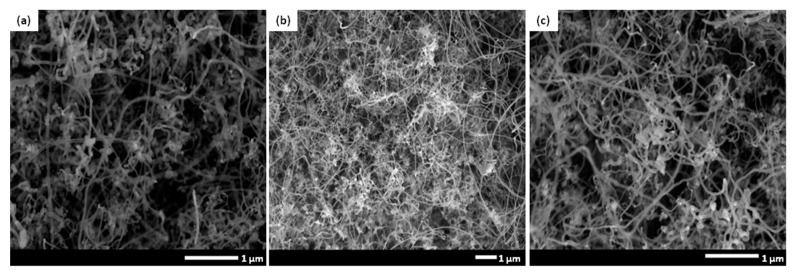
FE-SEM images of CNFs prepared with different ferrocene concentrations: (**a**) ferrocene (1.00 g), (**b**) ferrocene (2.21 g), (**c**) ferrocene (3.00 g).

**Figure 7:**
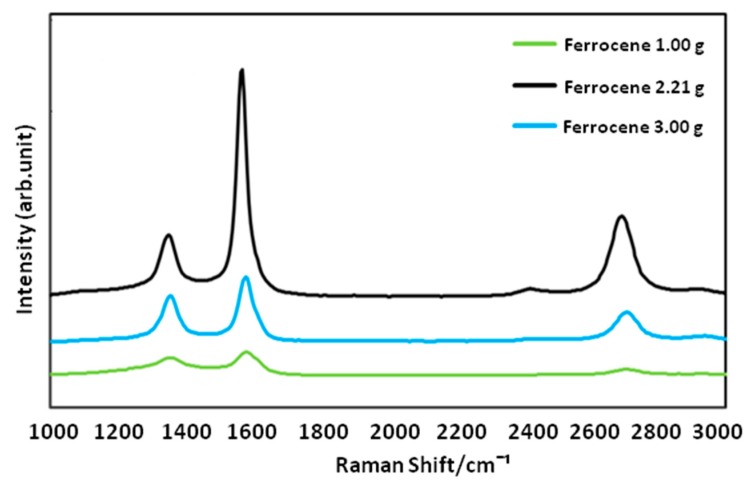
Raman spectra of CNFs prepared with different ferrocene concentrations.

It is highly known that presence of a proper catalyst plays a crucial role in achieving an optimized CNFCM with remarkable characteristics. Ferrocene, with the chemical formula of Fe(C_5_H_5_)_2_, is a relatively volatile organo–metallic compound with excellent vaporization above 400 °C. The molecular structure of ferrocene is broken up over high reaction temperature, resulting in the release of iron atoms. These iron atoms may later be agglomerated to form nanoparticles and speed up the growth rate of the CNFs. The influence of different ferrocene concentrations (1.00, 2.21, and 3.00 g) on the synthesis of CNFs is shown in Figure 6a–c. According to FE-SEM images, the CNFs fabricated with higher ferrocene concentrations (2.21 and 3.00 g) were denser and more aligned. Moreover, the presence of the aligned CNFs bundles clearly proved that the aggregation of the catalyst particles did not result in the coalescence of the catalyst particles, and the particles remain highly active. Therefore, a higher concentration of ferrocene was favorable for the growth of denser and more aligned CNFCMs. The Raman spectra of CNFs prepared with different ferrocene concentrations is illustrated in Figure 7.

### 3.5. Textural Properties of the Adsorbent

**Figure 8:**
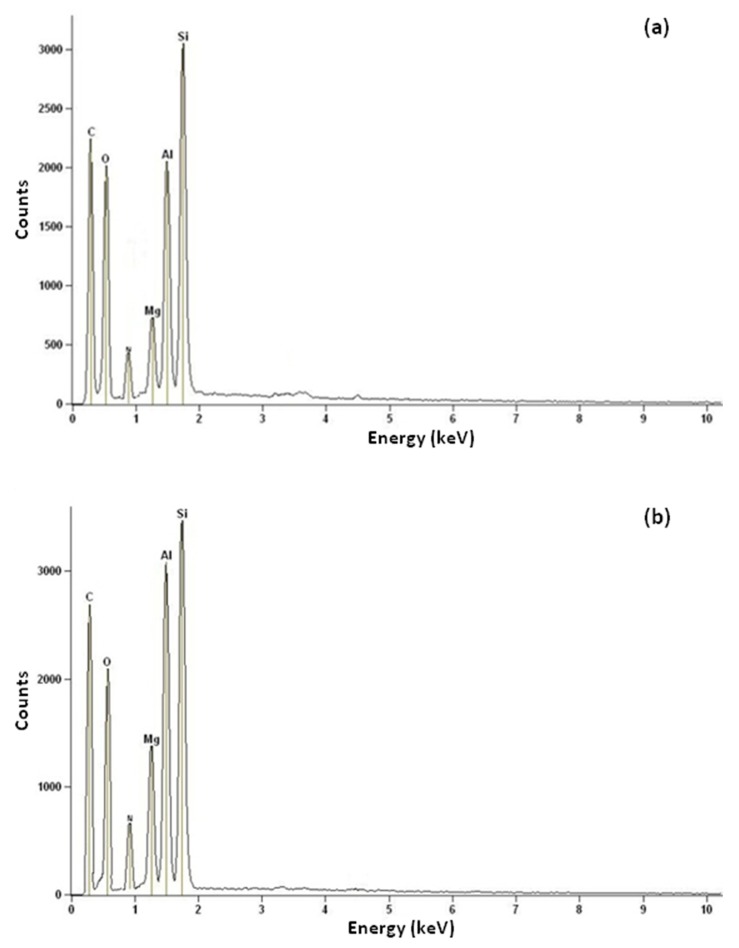
EDX analysis of (**a**) CNFCM and (**b**) modified CNFCM.

The elemental compositions studied using EDX as the compositional analyses are given in Table 6 and Figure 8. The results admitted the existence of C, O, Mg, Al, and Si in the CNF-coated monolith. The post-ammonia treatment was performed to provide the N_2_-rich-CNF-coated monolith. It was observed that the treated samples contained 2.24 wt% nitrogen groups.

**Figure 9:**
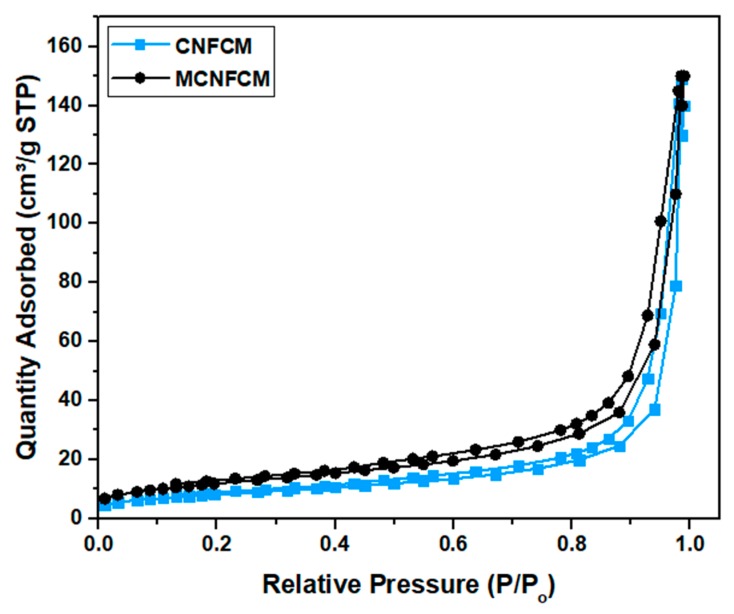
N_2_ ads–des isotherm of CNFCM and modified CNFCM.

Nitrogen ads-des isotherms (see Figure 9) were introduced to ascertain the S_BET_, total pore volume, and average pore size (presented in Table 7). The CNFCMs revealed II-type isotherms in line with IUPAC categorization, implying the mostly microporous characteristics of the sample. According to the results, N_2_ adsorption did not come into view at a low pressure (P/P_o_ < 0.5), indicating a weak adsorption of N_2_ for the mesostructured samples. On the other hand, N_2_ adsorption/desorption overlapped as a hysteresis loop at P/P_o_ > 0.5, which implied mesopore formation [21,22,23]. It is noteworthy that the monolith does not contribute to the S_BET_, and the reported values only represent the S_BET_ for the deposited carbon on the monolith. 

**Figure 10:**
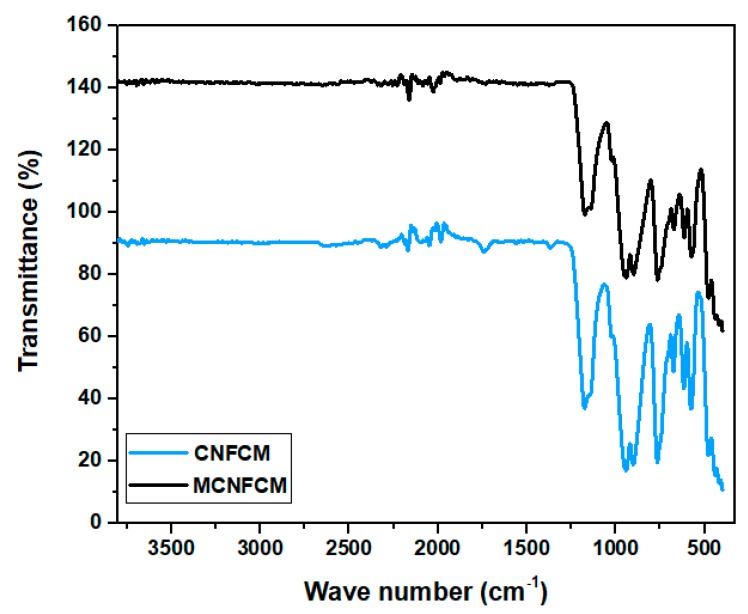
FTIR spectrums of CNFCM and modified CNFCM prepared over the following reaction conditions: reaction temperature of 800 °C, furfuryl alcohol flow of 0.08525 mL/min, ferrocene catalyst concentration of 2.21 g.

The presence of the different functional specious were verified via FTIR analyser, shown in Figure 10. The spectrum showed that the adsorbents limited a sequence of diverse functional specious. The peaks at 700, 750 and 1330 cm^−1^ might be attributed to the C-H bands. The peaks at 1200, 1550 and 2240 cm^−1^ corresponded to C-N, N-H and nitrile C≡N bands, respectively. The existence of these vibrations and bands proved that the post-ammonia treatment launched some N_2_-functional species onto the surface [24]. This implies that that the ammonia mixture bonded with the beads’ surface molecules to develop N_2_-covering surface-functional groups and active sites, hence improving the adsorption of CO_2_. The FTIR vibration at 3448 cm^−1^ corresponded to the bending vibrations of OH-functional groups. The FTIR spectrums of CNFCM indicated that the most important absorption bands, i.e., 1430, 833, and 715 cm^−1^, were correlated with the asymmetric vibration.

### 3.6. CO_2_ Adsorption Capability of CNF Coated Monolith

The adsorption experiments on CNFCF are usually done in various environmental conditions to examine the effect of some parameters during CO_2_ adsorption. In this research work, a flow-rate ranging 50–90 mL/min, adsorption temperature in the range 30–50 °C, different feed concentrations of CO_2_ in a range of 10%–40%, and a pressure from 1 to 2 bars were carried out to indicate the effect on adsorption capacity for each parameter.

### 3.7. Influence of Flow-Rates

**Figure 11:**
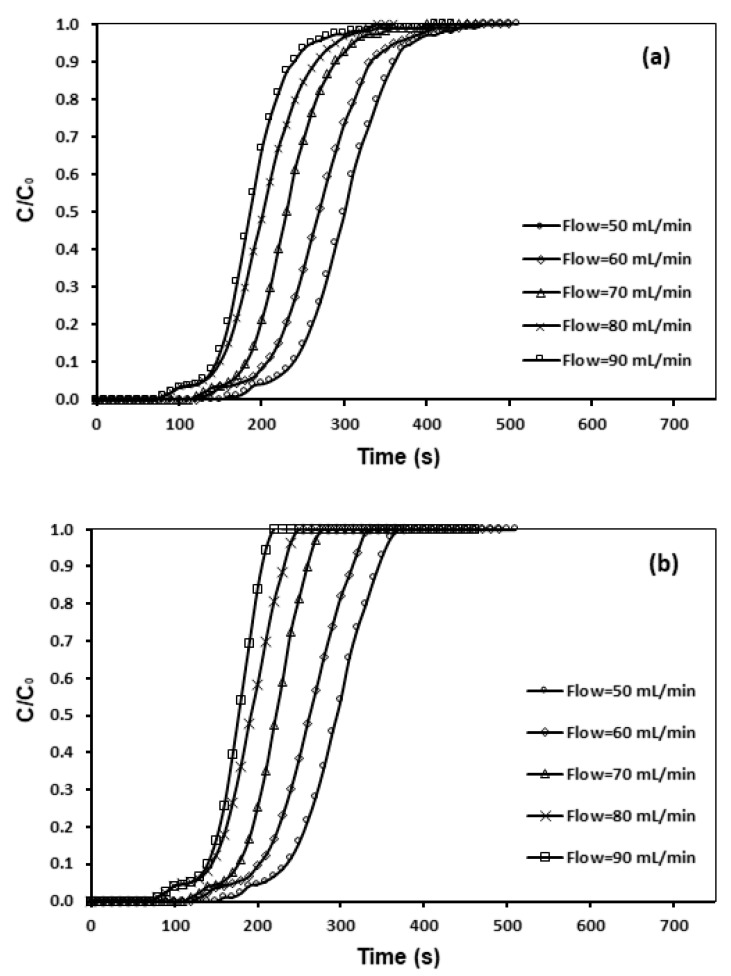
Breakthrough curve and effect of flow rate of (**a**) CNFCM and (**b**) modified CNFCM in constant T = 30 °C, P = 1 bar and 10% CO_2._

The influence of various flow rates on CO_2_ adsorption were examined, as shown in Figure 11, and Table 8. According to Garcia [25], at a higher feed flow rate, the adsorbate left behind the column prior to symmetry happens, which shows a shorter time required for breakthrough. Figure 11 revealed that, through escalating the flow rate from 50 to 90 mL/min, the breakthrough curve became sharper. The breakpoint time lessened from 470 to 190 s, whereas a longer time span was required for lower flow rates. This might be associated with the residence-time of the gas in the column, which was not prolonged sufficiently for adsorption symmetry to be attained at an excessive flow-rate. In addition, a preset saturation ability of the bed in accordance with the same driving force gave rise to a shorter time for saturation at an elevated flow-rate [18,19,26].

### 3.8. Effect of Feed Concentrations

**Figure 12:**
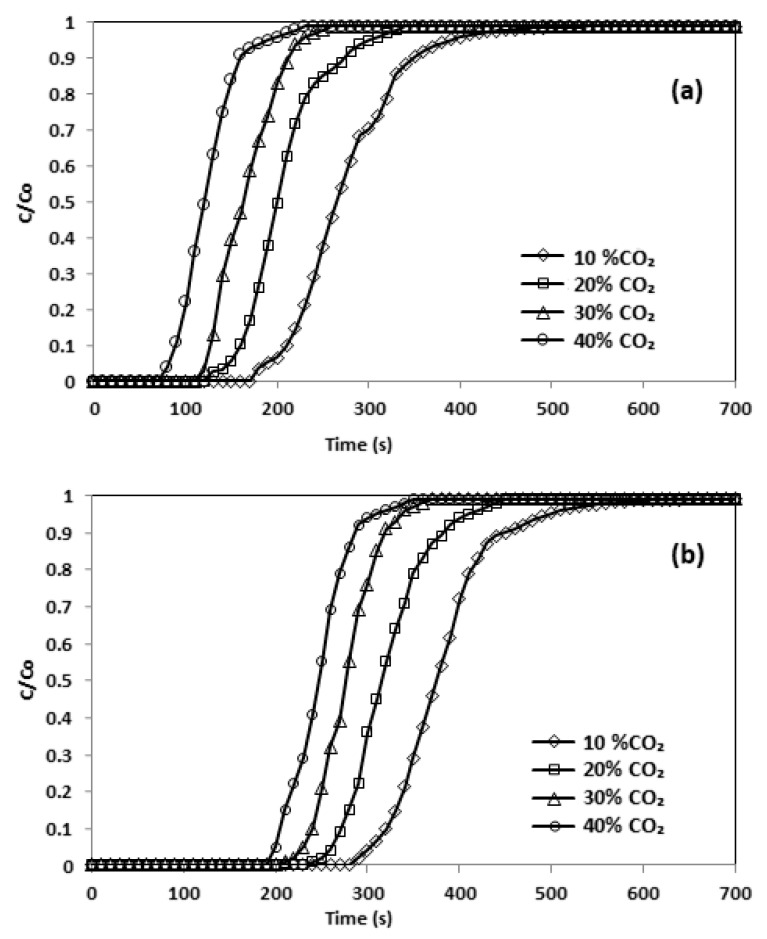
Breakthrough curve and effect of initial concentration (**a**) CNFCM and (**b**) modified CNFCM in constant T = 30 °C, P = 1 bar and Flow = 50 mL/min.

In accordance with Duran [18,19], at a higher feed intensity a sharp breakthrough curve is likely, since there should be a lower mass fluidity from the bulk to the surface of particles. The effects of the inlet adsorbate volume on effluent volume are shown in Figure 12. During these experiments, the flow rate was kept constant at 50 mL/min. This indicated that a longer time was required to reach C/C_0_ =1.0 for a lower inlet concentration of CO_2_. As estimated, at a lower concentration of CO_2_, longer reaction times were necessary for the effluent concentration to attain an amount equivalent to the inlet concentration. The breakpoint time dwindled from 350 to 110 s. The initial concentration of CO_2_ affected the uptake rate, which was improved by increasing the inlet concentration [26]. It can be observed that the adsorption of CO_2_ amplified from 0.23 to 2.22 mmol/g by raising CO_2_ concentration (Table 9).

### 3.9. Effect of Adsorption Temperatures

**Figure 13:**
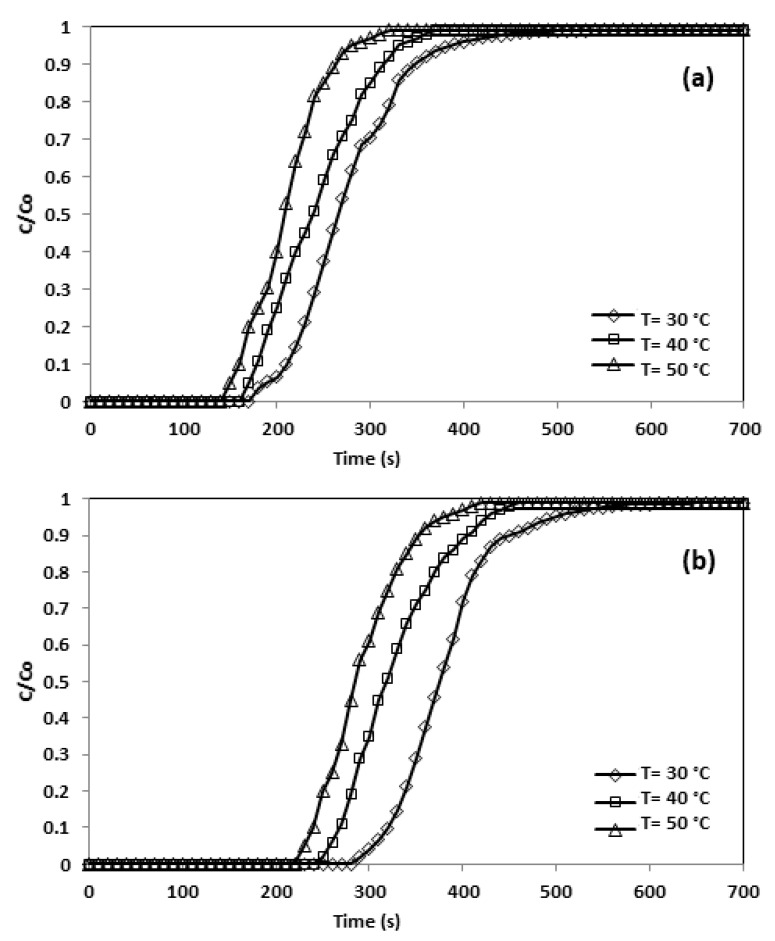
Breakthrough curve and effect of temperature (**a**) CNFCM and (**b**) Modified CNFCM in constant P = 1 bar, and 10% CO_2._

As can be seen in Figure 13, the sum of CO_2_ adsorbed lessened with escalating adsorption temperatures as a result of the physical characteristics of the adsorption procedure, and the lower the temperature, the larger the amounts of CO_2_ adsorbed. The exothermic quality of physisorption indicates that physical adsorption dominated the CO_2_ adsorption in CNFCM. In addition, the rigorous vibration of the molecules of gas caused by the elevated temperature made them complicated through the adsorption process [27]. Table 10 shows the adsorption decreased from 2.22 to 1.38 mmol/g as temperature rose.

### 3.10. Effect of Adsorption Pressures

**Figure 14:**
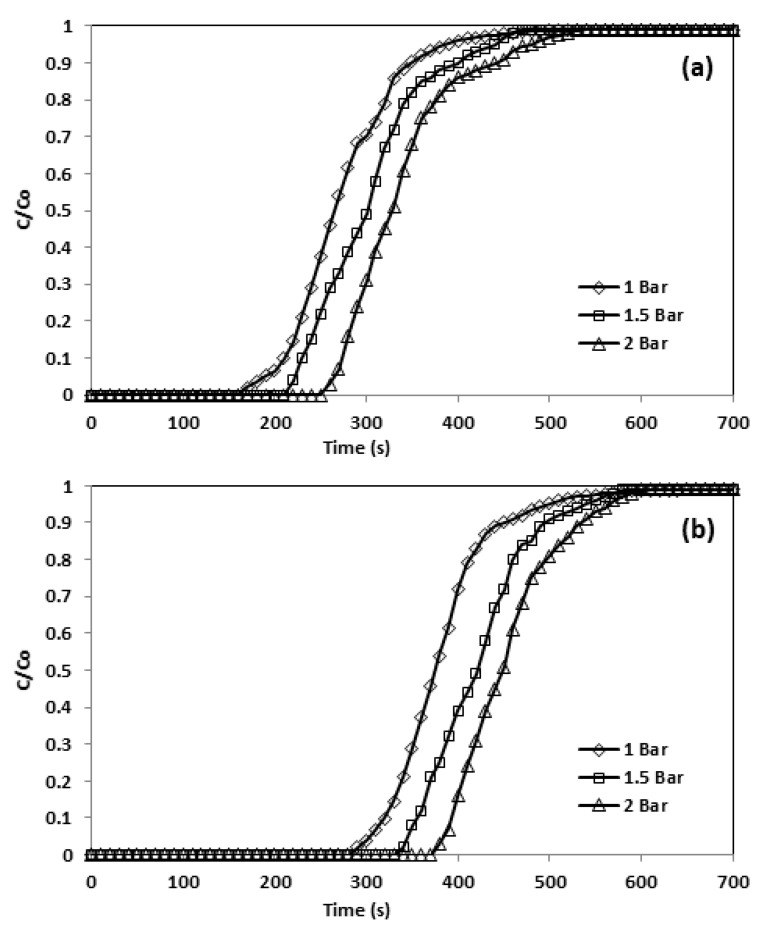
Breakthrough curve and effect of pressure (**a**) CNFCM and (**b**) modified CNFCM in constant T = 30 °C, Flow = 50 mL/min, and 10% CO_2._

In the instance of physisorption of gases over solids, the level of adsorption was enhanced by boosting the pressure of inlet CO_2_ (Table 11) as the volume of the gases decrease during adsorption. Figure 14 illustrates the pressure effect on adsorption capacity and breakthrough curve, where a higher adsorption pressure led to extended breakthrough times, as the CO_2_ concentration front took a longer time to reach the bed outlet. For instance, at 30 °C and 10% CO_2_ in the feed, the CO_2_ adsorption front reached the bed outlet following 250 s at 1 bar, and this time increased to 340 s at 2 bar.

### 3.11. Comparison of CO_2_ Adsorption on CNFCM and Modified CNFCM

The treated adsorbents have been tested as promising candidates for CO_2_ adsorption [18,19,26,28]. By tailor-made surface functionalization, the synthesized sorbents successfully adsorbed a large sum of CO_2_ via chemisorption. The extra N_2_ groups in NH_3_ were believed to play the role of active sites for the adsorption of CO_2_ as an acid gas. 

**Figure 15:**
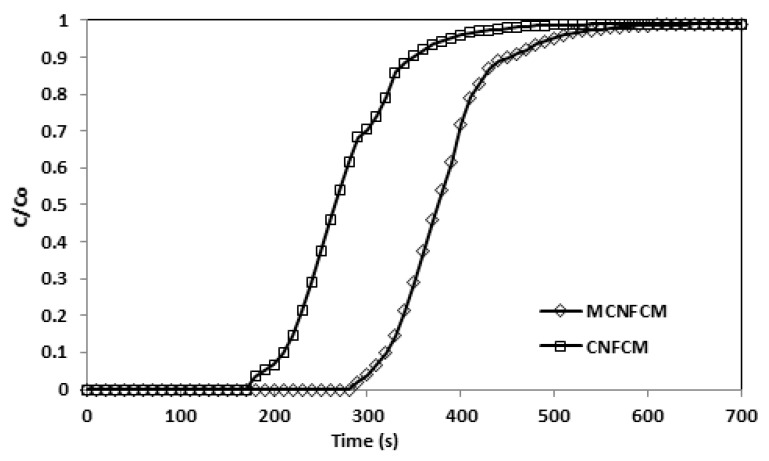
Breakthrough curves adsorption of CNFCM and modified CNFCM in constant 40% CO_2_, T = 30 °C, P = 1 Bar.

Figure 15 demonstrates that the modified CNFCM possessed superior adsorption volumes of CO_2_ as compared to CNFCM up to 46% (Table 12). Subsequent to surface treatment, the adsorption volumes of the MCNFCM for CO_2_ further improved. Therefore, the NH_3_-treatment raised a competition between expanding the chemical interaction by increasing N_2_ against lessening the physical interaction, since the pores were being broadened. Because the adsorption energies continuously increase in intensity, the chemical interaction should grow more rapidly than the physical portion, which is lessening. For the CNFCM, the capture of CO_2_ with an uncontaminated physisorption procedure was suggested as a monitoring process. The adsorptions of CO_2_ on the NH_3_-modified beads were supposedly the consequence of both the chemical adsorptions onto the N_2_ surface specious and physical adsorption in the pore channels.

### 3.12. Deactivation Model

To explore CO_2_ adsorption, the DM was utilized to model the breakthrough curves [25]. This model can analyze the breakthrough easily and correlated with adsorption isotherm. This simplifies the mathematical model and was found to be adequate when correlating the kinetic data. According to Equation (4), $\ln\left[\ln\left(\frac{C}{C_{0}}\right)\right]$ is plotted versus time. The parameters of deactivation model (k_d_, and k_s_α) can be obtained from the slope and intercept of the graph. The deactivation model parameters in 10% CO_2_ inlet were calculated and summarized in Table 13. As can be seen, the deactivation mode fit the adsorption data of with coefficients of determination (R^2^) of about 0.99.

### 3.13. Cyclic Regeneration

**Figure 16:**
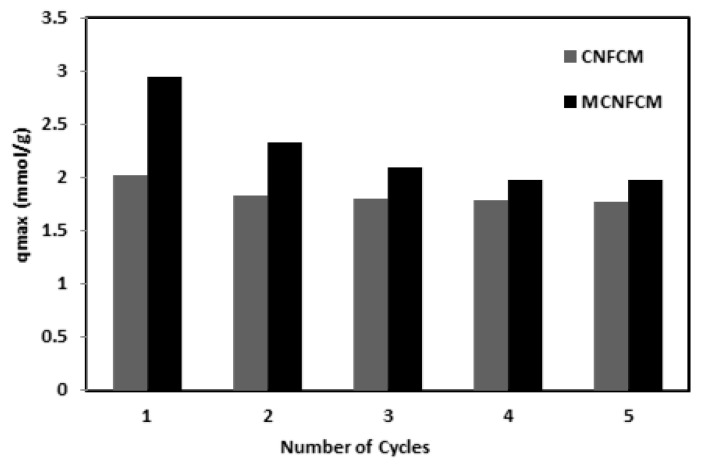
Cyclic adsorption of CO_2_ of CNFCM and MCNCM (40% CO_2_, T = 30 °C, P = 1 Bar).

A comparison study of the CO_2_ breakthrough volumes of CNFCM and MCNFCM are shown in Figure 16. Adsorption breakthrough experimentations were performed over the same terms, including a feed composition of 40%, temperature of 30 °C, pressure of 1 bar, and feed flow-rate of 50 mL/min. Figure 11 demonstrates the outcomes of five consecutive CO_2_ breakthrough reactions with CNFCM and MCNFCM. Moreover, the results exhibited a comparatively high drop in adsorption capability at modified samples in the second cycle. The decrease in adsorption capacity gradually slowed and became constant. This was distinguished as the inadequate desorption of CO_2_ in MCNFCM at 80 °C. The chemical interactions among segments of CO_2_ adsorbed in the pore channels of the MCNFCM treated with NH_3_ were solid; therefore, segments of CO_2_ could not be desorbed, causing a reduction in CO_2_ adsorption volume [28].

## 4. Conclusions

In this research work, the CNFs were formed over a monolith substrate by decomposition in presence of catalyst promoter. The four-level factorial design in RSM was employed to identify the key important factors on the fabrication process of the CNFCM. The fabricated CNFCMs had a S_BET_ of 95.38 m^2^/g and a total pore volume of 0.023 cm^3^/g. Based on the experiment results, the optimum reaction condition was at operating temperatures of 800 °C, a ferrocene catalyst concentration of 2.21 g, and a furfuryl alcohol flow-rate of 0.08525 mL/min. Moreover, the CNFs-coated monolith composite synthesized under optimum reaction conditions possessed a high degree of graphitic structure, which confirmed the growth of extremely pure and aligned CNFCMs. The carbon nanofiber-coated monolith was then modified by ammonia in order to evaluate the adsorption volume of CO_2_. Breakthrough curves relating to a diversity of reaction terms were evaluated. Shorter breakthrough time periods were gained, while gas flow-rate and the temperature were increased. On the other hand, a higher amount of CO_2_ and a higher pressure caused lengthier breakthrough periods. Moreover, the post-modification of as-synthesized CNFCM over NH_3_ treatment increased the intensity of functional groups and consequently improved the adsorption capability of CO_2_. The ammonia treatment can introduce some N_2_-containing functional specious onto the surface of CNFCM of up to 2.24 wt.%. The presence of N_2_- and O_2_-containing functional specious on the surface of the CNFCM caused the enhancement of the microporosity of the synthesized CNFCM. Furthermore, a significant enhancement in adsorption capacity (above 46%) was observed with the modified sample, implying that the existence of surface functional groups could be even more influential than porosity during the adsorption process.

## Figures and Tables

**Table 1:** Response surface methodology (RSM) experimental factors and levels.

Variables	Coded Level of Variables		
	−1	0	1
A: Temperature [°C]	700	800	900
B: Catalyst [g]	1	2	3
C: Hydrocarbon flow rate [mL/min]	0.0750	0.0875	0.100
D: Cat promoter [g/mL]	0.050	0.125	0.200

**Table 2:** Experimental matrix of four-level factorial plan in the RSM system.

Run	Factors				Response	
	A	B	C	D	CNF Yield (%)	
	Temperature [°C]	Catalyst [g]	Hydrocarbon Flow [mL/min]	Catalyst Promoter [g/mL]	Actual	Predicted
1	900	2	0.0750	0.125	16.4596	16.9256
2	800	2	0.0875	0.125	15.2727	15.2232
3	800	2	0.0875	0.125	14.5475	15.2238
4	800	2	0.0750	0.050	14.8155	15.2464
5	700	2	0.0750	0.125	12.1616	11.6175
6	800	3	0.0875	0.050	16.5361	15.9834
7	800	2	0.1000	0.050	15.1856	15.1619
8	700	3	0.0875	0.125	12.3192	12.9016
9	800	1	0.0875	0.200	14.8515	14.6116
10	800	3	0.1000	0.125	16.443	16.9681
11	700	2	0.0875	0.050	12.4124	12.6834
12	800	1	0.0875	0.050	13.7732	13.7227
13	900	1	0.0875	0.125	14.7475	14.6504
14	700	1	0.0875	0.125	12.7677	12.8704
15	800	2	0.1000	0.200	15.8248	15.8858
16	900	2	0.1000	0.125	15.6111	15.3792
17	900	2	0.0875	0.200	15.321	15.3705
18	800	3	0.0875	0.200	15.4059	14.6603
19	800	1	0.1000	0.125	14.2424	14.7262
20	900	3	0.0875	0.125	16.545	16.9301
21	800	2	0.0875	0.125	14.899	15.2237
22	700	2	0.0875	0.200	12.1188	12.4808
23	800	2	0.075	0.200	13.5842	14.1089
24	800	2	0.0875	0.125	15.4747	15.2256
25	700	2	0.1000	0.125	15.6263	14.8709
26	800	3	0.0750	0.125	15.1919	15.0338
27	800	1	0.0750	0.125	15.115	14.9356
28	900	2	0.0875	0.050	15.651	15.6043
29	800	2	0.0875	0.125	15.8899	15.2234

**Table 3:** Chronological model summation of squares.

Source	Summation of Squares	DF	Mean Square	F Value	Prob > F	R^2^
Linear	31.91415553	4	7.978539	9.550314	0.0491	0.6026
2FI	10.92041913	6	1.820070	3.588426	0.0365	0.7983
Quadratic	4.875996848	4	1.218999	4.012031	0.0152	0.9117
Cubic	1.429150224	8	0.178644	0.37948	0.8817	0.9427

**Table 4:** The analyses of variance (ANOVA) for carbon yield response surface quadratic model.

Source	Sum of Squares	DF	Mean Square	F Value	Prob > F
A	25.31	1	25.31	76.46	< 0.0001
B	4.02	1	4.02	12.14	0.0037
C	2.17	1	2.17	6.56	0.0226
D	0.13	1	0.13	0.4	0.5351
AB	1.26	1	1.26	3.81	0.0713
AC	5.79	1	5.79	17.49	0.0009
AD	0.032	1	0.032	0.01	0.9752
BC	1.13	1	1.13	3.41	0.0862
BD	1.22	1	1.22	3.68	0.0756
CD	0.87	1	0.87	2.64	0.1264
A2	4.1	1	4.1	12.39	0.0034
B2	0.045	1	0.045	0.14	0.7182
C2	0.47	1	0.47	1.42	0.2525
D2	0.99	1	0.99	2.99	0.1059
Residual	4.64	14	0.33	—	—

**Table 5:** Predicted results by RSM.

Source	Temperature (°C)	Catalyst (g)	Hydrocarbon Flow Rate (mL/min)	Catalyst Promoter (g/mL)	CNF Yield Predicted (%)	CNF Actual Yield (%)	RSE (%)
Condition 1	800	2.21	0.085	0.095	16.975	16.505	2.77
Condition 2	838	2.97	0.098	1.154	16.969	16.401	3.35
Condition 3	889	2.30	0.077	0.092	16.965	16.411	3.27

**Table 6:** EDX analysis of CNF-coated monolith and the modified CNF-coated monolith.

Elements	Weight (%)	
	CNFCM	MCNFCM
C	53.24	54.46
O	37.33	35.29
Mg	2.15	1.84
Al	2.56	2.99
Si	3.01	3.11
N	1.71	2.31

**Table 7:** N_2_ Ads–des analysis of CNFCM and modified CNFCM.

Sample	S_BET_ (m^2^/g)	Pore Volume (cm^3^/g)		Pore Size (nm)
		Micropore Volume	Mesopore Volume	
CNFCM	95.38	0.023	0.164	1.95
MCNFCM	98.70	0.023	0.160	1.98

**Table 8:** CO_2_ adsorption over different flow rates.

CO_2_ Flow Rate (mL/min)	q_max_ (mmol/g)	
	CNFCM	MCNFCM
50	0.58	0.65
60	0.67	0.71
70	0.73	0.84
80	0.82	0.93
90	0.89	1.01

**Table 9:** CO_2_ adsorption over different initial concentrations.

CO_2_ Concentrations (%)	q_max_ (mmol/g)	
	CNFCM	MCNFCM
10	0.89	1.01
20	1.33	1.53
30	1.94	2.12
40	2.22	2.94

**Table 10:** CO_2_ adsorption over different temperatures.

Temperature °C	q_max_ (mmol/g)	
	CNFCM	MCNFCM
30	2.22	2.94
40	1.96	2.65
50	1.38	2.53

**Table 11:** CO_2_ adsorption over different pressures.

Pressure (bar)	q_max_ (mmol/g)	
	CNFCM	MCNFCM
1.0	2.02	2.94
1.5	2.69	3.05
2.0	2.95	3.11

**Table 12:** CO_2_ adsorption of CNFCM and modified CNFCM.

Adsorbent	q_max_ (mmol/g)
CNCFM	2.02
MCNFCM	2.94

**Table 13:** Deactivation model parameters in 10% CO_2_ inlet.

Adsorbent	F (mL/min)	T (°C)	P (bar)	k_d_ (s)	k_s_α (-)	R^2^
CNFCM	50	30	1	0.0392	10.729	0.981
MCNFCM	50	30	1	0.0398	10.812	0.991
